# Incidence of traumatic sciatic nerve injury in patients with acetabular fractures and factors affecting recovery: a retrospective study

**DOI:** 10.1186/s13018-023-03515-z

**Published:** 2023-01-12

**Authors:** Zhigang Liu, Fulin Tao, Weicheng Xu, Fanxiao Liu, Jinlei Dong, Lianxin Li, Zhenhai Hao, Dongsheng Zhou, Shun Lu

**Affiliations:** 1 Department of Orthopaedics Surgery, Haining People’s Hospital, Jiaxing City, Zhejiang Province China; 2 Department of Orthopaedics Surgery, Shandong Provincial Hospital affiliated to Shandong First Medical University, Jinan City, Shandong Province China; 3 Shandong Trauma Center, Jinan City, Shandong Province China

**Keywords:** Acetabular fractures, Traumatic sciatic nerve injury, Nerve recovery

## Abstract

**Background:**

Reports on traumatic sciatic nerve injury associated with acetabular fracture are rare. In this study, we investigated the demographics of these injuries, their clinical characteristics, management, and factors potentially influencing neurological recovery.

**Methods:**

We retrospectively reviewed all patients diagnosed to have acetabular fracture at our trauma center between January 2014 and June 2021. Data on patient demographics, characteristics of sciatic nerve injury, neurological recovery, factors potentially influencing neurological recovery were analyzed.

**Results:**

Eighteen patients (bilateral in one case) met the diagnostic criteria. All these injuries involved the posterior wall or posterior column, and most patients had posterior dislocation of the hip joint. Four of the 19 sides with traumatic sciatic nerve injury involved the common peroneal nerve division and 15 involved both the common peroneal and tibial nerve divisions. Seventeen patients (18 sides) underwent intraoperative nerve exploration, which revealed abnormalities in 7 sides and no obvious abnormality in 11 sides. At the last follow-up, 10 sides (52.6%) had complete recovery and 9 (47.4%) had partial recovery; the difference was statistically significant between those with or without abnormal nerve damage during exploration (*P* = 0.046). Linear regression analysis showed that a nerve abnormality detected intraoperatively was a predictor of nerve recovery (*P* = 0.009). The mean recovery time was significantly longer for partial recovery than for complete recovery (13.78 months vs. 6.70 months; *P* = 0.001).

**Conclusions:**

All the injuries in this series involved the posterior wall or posterior column, and most patients had posterior dislocation of the hip joint. Damage to the common peroneal nerve division was more severe than that to the tibial nerve division preoperatively. However, the degree of recovery of the common peroneal division was not worse than that of the tibial division. There was a relationship between the degree of neurological recovery and whether there was an abnormality at the time of intraoperative nerve exploration. Patients with partial recovery took longer to recover.

## Background

Acetabular fracture is a severe intra-articular injury and is usually caused by high-energy trauma, such as a road traffic accident or a fall from height [1–4]. Most acetabular fractures are associated with polytrauma involving the brain, thorax, abdomen, extremities, or other sites. Traumatic sciatic nerve damage is one of the most serious injuries associated with acetabular fracture [5, 6].

The sciatic nerve is the largest and longest nerve in the body. It is the motor nerve of the hamstrings and calf and foot muscles and is an important sensory nerve of the calf and foot. The sensory and motor functions of almost all areas below the knee are innervated by the sciatic nerve [7, 8]. This nerve is highly susceptible to injury as a result of displaced acetabular fracture or a dislocated femoral head. Incorrect diagnosis and treatment may seriously affect lower extremity function [9, 10].

Acetabular fracture combined with sciatic nerve damage has an incidence of about 3.3–33% [5, 11, 12]. A significant proportion of traumatic sciatic nerve injuries recover spontaneously with favorable outcomes [13, 14]. Although a detailed preoperative clinical examination is important for detection of sciatic nerve injury, abnormal appearance of the sciatic nerve can be confirmed by the surgeon intraoperatively, which facilitates diagnosis and treatment [15, 16]. However, there are no relevant reports on the indications for surgical treatment of traumatic sciatic nerve injury or its timing. At present, there is scant literature on traumatic sciatic nerve injury in association with acetabular fracture, and the available research involves small sample sizes and is mainly focused on the incidence and characteristics of this nerve injury [17]. Moreover, there are no studies on neurological recovery or the factors affecting neurological recovery.

This retrospective study reviewed the patient characteristics, clinical features, and management of traumatic sciatic nerve injury associated with acetabular fracture and evaluated the characteristics of neurological recovery from this nerve injury and the factors potentially influencing recovery.

## Methods

We retrospectively reviewed the patients with a diagnosis of acetabular fracture with traumatic sciatic nerve injury managed in our trauma center between January 2014 and June 2021. All patients were under the care of the Shandong Provincial Hospital Affiliated to Shandong First Medical University. All patients underwent procedures performed by a same surgical team. The study was approved by the Medical Ethical Committee at the authors’ institution. The present study conforms to the Declaration of Helsinki. All patients involved gave informed consent and all data were anonymized before the analysis to safeguard patient privacy.

### Data collection

Information was collected on age, sex, mechanism of injury, Injury Severity Score (ISS), Abbreviated Injury Scale (AIS) score, associated injuries, type of fracture according to the Letournel-Judet classification system [18], whether or not the hip was dislocated, type of sciatic nerve injury, degree of nerve injury according to the Louisiana State University Health Science Center (LSUHSC) peripheral nerve score [9], and degree of neurological recovery (none, partial, or complete according to the classification criteria devised by Natasha et al. [5]).

### Statistical analysis

All statistical analyses were performed using SPSS software version 24.0 (IBM Corp., Armonk, NY, USA). Data on parameters such as age, ISS, AIS, LSUHSC peripheral nerve score and recovery time were described using the mean ± standard deviation and *t*-tests were used for comparison between groups. The skewness and kurtosis, graphical methods, and nonparametric tests were used for normality tests, and the Mann Whitney test was used for nonparametric comparison of continuous variables. The categorical variable data that affected the likelihood of traumatic sciatic nerve injury were analyzed using Fisher’s exact test and the chi-square test. Linear regression analysis was used to determine whether the presence or absence of intraoperatively nerve damage predicted the degree of neurological recovery. A *P*-value of < 0.05 was considered statistically significant.

## Results

### General information

A total of 195 patients (204 sides) had complete clinical and radiographic data and were eligible for inclusion in the study. Type of fracture was described using the Letournel-Judet classification system. Patients with spinal cord injury (*n* = 7, 8 sides) or iatrogenic sciatic nerve injury (*n* = 2, 2 sides) were excluded, leaving 186 patients (194 sides) with acetabular fracture, 18 (9.7%) of whom sustained traumatic sciatic nerve injury, which was bilateral in one case (Table 1).

**Table 1:** Demographic information, trauma scoring, Mechanism of injury and additional injuries

Characteristics	Mean (± SD)/No. of Patients(*n* = 18)	Range/Percentage
Age (y)	43.1 ± 16.1	23–69
ISS	32.5 ± 13.0	17–57
AIS	20.4 ± 8.5	9–41
No. sex	11 (61.1%) male, 7 (38.9%) female	
*Mechanism of injury*		
Motor vehicle collision	11	61.1%
Fall from height	3	16.7%
Struck by falling objects	2	11.1%
Fall from bicycle	1	5.6%
Truck crush	1	5.6%
Hemorrhagic shock	10	55.6%
*Additional injuries*		
Head	3	16.7%
Chest	8	44.4%
Spine	3	16.7%
Abdomen	7	38.9%
Pelvic cavity	3	16.7%
Upper extremity	6	33.3%
Ipsilateral lower extremity	4	22.2%
Contralateral lower extremity	0	0
Bilateral lower extremity	4	22.2%

The 18 patients with acetabular fracture and traumatic sciatic nerve injury included 11 men and 7 women with a mean age of 43.1 years (range, 23–69). The average ISS was 32.5 (range, 17–57) and the average AIS score was 20.4 (range, 9–41). The main cause of injury was a road traffic accident in 11 cases, a fall from height in 3, and a falling object in 2; 55.6% of the patients sustained hemorrhagic shock. Associated injuries were common, in particular those involving the chest and abdomen, as were fractures of a lower extremity (Table 1).

According to the Letournel-Judet classification system, 2 sides had posterior wall fractures, 2 had posterior column fractures, 2 had transverse fractures, 3 had T-shaped fractures, 1 had posterior wall and column fractures, 3 had transverse and posterior wall fractures, 1 had anterior column and posterior hemitransverse fractures, and 5 had fractures in both columns. Nine sides were associated with posterior dislocation of the hip joint, 2 with central dislocation and 2 with fracture of the femoral head (Table 2).

**Table 2:** Fracture pattern and related injuries for traumatic sciatic nerve

	No. of traumatic sciatic nerve injury (*n* = 19)	Percentage
*Classification*		
Posterior wall	2	10.5%
Posterior column	2	10.5%
Anterior wall	0	0
Anterior column	0	0
Transverse	2	10.5%
T-type	3	15.8%
posterior column and posterior wall	1	5.3%
Transverse and posterior wall	3	15.8%
Anterior column posterior hemitransverse	1	5.3%
Associated both column	5	26.3%
*Related injuries*		
Posterior dislocation	9	47.4%
Central dislocation	2	10.5%
Femoral head fracture	2	10.5%

### Preoperative traumatic sciatic nerve injury

Four (21.1%) of the 19 traumatic sciatic nerve injuries involved the common peroneal nerve division and 15 (78.9%) involved both the common peroneal nerve division and the tibial nerve division. There were no cases of isolated tibial nerve division injury (Table 3).

**Table 3:** Main characteristics and data of 19 acetabular fractures with sciatic nerve injury

	Sex	Age (years)	Fracture classification	Surgical approach	type of nerve injury	Observation	Preoperative scoring	Follow-up scoring			Recovery Time (months)	Follow-up time (months)
							Peroneal nerve	Tibial nerve	peroneal nerve	Tibial nerve		
1	M	55	Both column	Anterior/ posterior	Both divisions	Contusion	2	2	3	4	14	95
2	F	23	Both column	Anterior/ posterior	Peroneal division	None	1	5	4	5	10	92
3	F	25	Both column	Anterior/ posterior	Both divisions	None	3	3	5	5	6	95
4	F	58	Transverse	Anterior/ posterior	Both divisions	None	1	1	5	5	8	86
5	M	51	Both column	Posterior	Peroneal division	None	3	5	5	5	3	96
6	M	60	Transverse and posterior wall	Anterior/ posterior	Both divisions	Compression	3	3	5	5	8	68
7	M	69	Anterior column posterior hemitransverse	Nonsurgical	Both divisions	Nonsurgical	2	2	5	5	7	69
8	F	29	Posterior column	Anterior/ posterior	Peroneal division	Compression	1	5	3	5	18	70
9	F	29	Both column	Anterior/ posterior	Both divisions	None	3	3	5	5	6	70
10	M	40	Transverse	Posterior	Peroneal division	None	2	5	4	5	23	65
11	M	35	Posterior wall	Posterior	Both divisions	None	2	3	5	5	5	54
12	M	28	T-shaped	Posterior	Both divisions	None	2	3	5	5	4	61
13	M	35	Transverse and posterior wall	Posterior	Both divisions	None	1	2	4	4	12	49
14	F	68	Posterior column	Posterior	Both divisions	None	1	1	5	5	12	31
15	M	29	Posterior wall	Anterior/ posterior	Both divisions	Traction	3	3	5	5	8	34
16	M	68	Posterior wall and posterior column	Posterior	Both divisions	Compression	0	0	1	2	6	28
17	M	48	Transverse and posterior wall	Anterior/ posterior	Both divisions	contusion	1	1	3	3	18	74
18	F	25	T-shaped	Posterior	Both divisions	Traction	1	1	2	2	9	74
19			T-shaped	Posterior	Both divisions	None	2	2	4	4	14	74

### Preoperative neurological function

The average LSUHSC peripheral nerve score was 1.79 ± 0.92 in patients with common peroneal nerve division injury and 2.68 ± 1.57 in those with tibial nerve division injury; the difference was statistically significant (*P* = 0.040) (Table 4).

**Table 4:** Preoperative and recovery neurological function

	Nerve division	Mean	Standard deviation	*P*
Preoperative	Peroneal division	1.79	0.92	0.040^&*^
	Tibial division	2.68	1.57	
Recovery	Peroneal division	4.11	1.20	0.387^&^
	Tibial division	4.42	1.02	
Peroneal division recovery	Preoperative	1.79	0.92	0.000^&***^
	Last follow-up	4.11	1.20	
Tibial division recovery	Preoperative	2.68	1.57	0.000^&***^
	Last follow-up	4.42	1.02	
Both divisions recovery	Preoperative	4.42	2.12	0.000^&***^
	Last follow-up	8.53	2.14	

^&^Student's *t* test

*P < 0.05, ***P < 0.001

### Intraoperative nerve observation

Seventeen patients (18 sides) underwent nerve exploration using the Kocher-Langenbeck approach. The exception was 1 patient (1 side) who was not a candidate for surgery because of poor health status. Intraoperative observation showed that 3 sides were compressed by bone fragments, 2 were stretched, 2 were contused, and 11 showed no obvious abnormality (Table 3).

### Recovery of neurological function

The median duration of follow-up was 70 months (range, 28–96). At the last follow-up, 10 sides (52.6%) with sciatic nerve injury had recovered completely, 9 sides (47.4%) had recovered partially, and no patient had no recovery. Nine of 19 common peroneal nerve division injuries showed partial recovery and 10 showed complete recovery; the complete recovery rate was 52.6%. Six of 15 tibial nerve division injuries recovered partially and 9 recovered completely, giving a complete recovery rate of 60%. The mean common peroneal nerve division score was 4.11 ± 1.20 and the mean tibial nerve division score was 4.42 ± 1.02. There were significant differences in the LSUHSC peripheral nerve score for the common peroneal nerve division, tibial nerve division, and common peroneal and tibial nerve divisions combined before surgery and at the last follow-up (*P* = 0.000) (Table 4).

At the final follow-up, the LSUHSC peripheral nerve score in the 18 patients who underwent intraoperative nerve exploration was 7.00 ± 3.61 for 3-sided nerve compression, 7.00 ± 4.24 for 2-sided nerve stretch, 6.50 ± 0.71 for 2-sided nerve contusion, and 9.45 ± 0.82 for the 11 nerves with no abnormality. There was no significant difference in the degree of neurological recovery between the above three groups in which a sciatic nerve abnormality was found during intraoperative exploration (*P* > 0.05). However, there was a statistically significant difference in neurological recovery between those with and without an intraoperative sciatic nerve abnormality (*P* = 0.046) (Table 5). Linear regression analysis showed that intraoperative detection of a nerve abnormality predicted nerve recovery (*P* = 0.009) (Table 6).

**Table 5:** Intraoperative nerve observation and the recovery neurological function

	Compression (*n* = 3)	Stretch (*n* = 2)	Contusion (*n* = 2)	No abnormallity(*n* = 11)
Mean (± SD)	7.00 ± 3.61	7.00 ± 4.24	6.50 ± 0.71	9.45 ± 0.82
*P*	0.218^&^	0.334^&^	0.188^&^	0.046^&*^

*SD* Standard deviation

^&^Student’s *t* test

*P < 0.05

**Table 6:** Linear regression analysis of intraoperative detection of a nerve abnormality

	Unstandardized coefficients		Standardized coefficients	*t*	Sig	Collinearity Statistics	
	B	Std. Error	Beta			Tolerance	VIF
Intraoperative detection of a nerve abnormality	2.597	0.868	0.599	2.992	0.009**	1.000	1.000
Comments	4.260	1.461		2.915	0.010		

*P < 0.05, **P < 0.01, ***P < 0.001

### Partial recovery vs complete recovery after sciatic nerve injury

The degree of recovery after sciatic nerve injury was not related to sex, age, cause of injury, type of fracture, dislocation of the hip joint, or injury to the femoral head (*P* > 0.05). However, patients in whom the injured nerve showed partial recovery had a significantly longer mean recovery time (13.78 months [range, 6–23] vs. 6.70 months [range, 3–12]; *P* = 0.001) (Table 7).

**Table 7:** Partial recovery and complete recovery after sciatic nerve injury

	Total (*n* = 19)	Partial recovery (*n* = 9)	Complete recovery (*n* = 10)	*P*
Age (years)	42.1 ± 16.2	38.7 ± 15.6	45.2 ± 17.8	0.408
Male	11 (57.9%)	5 (55.6%)	6 (60.0%)	1.000
*Mechanism of injury*				
Motor vehicle collision	11 (57.9%)	5 (55.6%)	6 (60.0%)	1.000
Fall from height	4 (21.6%)	2 (22.2%)	2 (20.0%)	1.000
Struck by falling objects	2 (10.5%)	1 (11.1%)	1 (10.0%)	1.000
Fall from bicycle	1 (5.3%)	1 (11.1%)	0 (0)	0.474
Truck crush	1 (5.3%)	0 (0)	1 (10.0%)	1.000
*Classification*				
Posterior wall	2 (10.5%)	0 (0)	2 (20.0%)	0.474
Posterior column	2 (10.5%)	1 (11.1%)	1 (10.0%)	1.000
Transverse	2 (10.5%)	1 (11.1%)	1 (10.0%)	1.000
Posterior column and posterior wall	1 (5.3%)	1 (11.1%)	0 (0)	0.474
Transverse and posterior wall	3 (15.8%)	2 (22.2%)	1 (10.0%)	0.921
T-type	3 (15.8%)	2 (22.2%)	1 (10.0%)	0.921
Anterior column posterior hemitransverse	1 (5.3%)	0 (0)	1 (10.0%)	1.000
Associated both column	5 (26.3%)	2 (22.2%)	3 (30.0%)	1.000
*Related injuries*				
Posterior dislocation	9 (47.4%)	5 (55.5%)	4 (40.0%)	0.827
Central dislocation	2 (10.5%)	1 (11.1%)	1 (10.0%)	1.000
Femoral head fracture	2 (10.5%)	1 (11.1%)	1 (10.0%)	1.000
Recovery Time (months)	10.05 ± 5.22	13.78 ± 5.26	6.70 ± 2.54	0.001**

*P < 0.05, **P < 0.01, ***P < 0.001

## Discussion

Sciatic nerve injury associated with acetabular fracture is most likely to occur with dislocation of the hip joint. Letournal and Judet found traumatic sciatic nerve palsy in 75% of patients with posterior dislocation of the hip but in only 22% of those with central dislocation [19]. Fassler et al. reported that the traumatic sciatic nerve injury rate associated with a displaced acetabular fracture was approximately 20% [12]. Natasha et al. found that the incidence of nerve injury with acetabular fracture was 3.3%, that 75% of the nerve injuries were caused by trauma, and that 23 of 24 traumatic nerve injuries were associated with posterior dislocation of the hip [5]. Our findings are similar to those of previous studies. However, a meta-analysis published in 2022 found that the incidence of post-traumatic sciatic nerve injury was 5.1% irrespective of type of fracture [17].

Letournel and Judet noted that the highest incidence of sciatic nerve palsy occurred in association with a posterior fracture dislocation of the hip joint [19]. Helfet and Schmeling found that all patients with post-traumatic sciatic nerve injury had a fracture pattern that included the posterior wall or column. The impact of these injuries may result in blunt contusion to the sciatic nerve, laceration, or stretching of the nerve over the dislocated femoral head [20]. Reports on surgical indications for traumatic sciatic nerve injury are rare. Recovery of nerve injury is closely related to the integrity of the nerve in patients who are treated surgically. The likelihood of recovery with good nerve integrity is highest after surgical release, intermediate after suturing, and less likely but possible after graft repair [9]. The success of surgical decompression for nerve damage depends on several preoperative factors, including the severity of the initial injury, and the intraoperative findings. There is some controversy regarding the timing of surgery according to whether the initial injury was traumatic or iatrogenic and management of late complications [20]. Unlike the other two types of nerve injury, sciatic nerve injury in a patient with an acetabular fracture often involves the posterior wall, posterior column, or posterior dislocation of the hip joint. Therefore, posterior surgery (mostly using the Kocher-Langenbeck approach) is required to reconstruct the acetabulum and femoral head and allows nerve exploration during open reduction or internal fixation of an acetabular fracture. There have been some rare reports on the appearance of the sciatic nerve at the time of surgery. In a study by Fasslers et al., the sciatic nerve was in continuity in all 14 patients, definite contusions were seen in 9, the nerve appeared to be normal in 3, and 2 nerves were visualized but the appearance was not mentioned in the operation report [12]. In the present study, intraoperative observation confirmed that all patients had full continuity of the nerve and that 3 sides were compressed by bone fragments, 2 were stretched, 2 were contused, and 11 had no obvious abnormality.

Recovery after a peripheral nerve injury has long been a difficult issue in clinical orthopedics, and the poor prognosis has caused considerable inconvenience. Studies on the natural history of sciatic nerve injury have yielded conflicting results. Letournel and Judet reported that 60% of sciatic nerve injuries showed significant recovery [21]. However, Tile noted that 75% of post-traumatic injuries and all iatrogenic injuries recovered either completely or partially [22]. In a recent study by Natasha et al., 50% of patients experienced partial nerve recovery and 22% had complete recovery [23]. A meta-analysis published in 2022 reviewed 20 studies that included 651 patients with 44 post-traumatic sciatic nerve injuries and reported complete recovery from nerve injury in 64.7% of cases [17]. In the present study, recovery of neurological function was very good and significantly improved at follow-up when compared with preoperative function. There were no cases in which there was no recovery; 52.6% had complete recovery and 47.4% had partial recovery. Two patients still required an ankle–foot orthosis at the last follow-up.

There is limited literature on the time required for an injured sciatic nerve to recover, and the few reports that are available are based on very small sample sizes. The time needed for complete sciatic nerve recovery in the literature varies from 1.5 months to 24 months [24–27]. In the study by Natasha et al., the median recovery time was relatively longer for patients with partial nerve recovery than for those with complete nerve recovery (27 months vs. 17 months) [5]. In our study, the mean recovery time was 10 months (range, 3–23) and was significantly longer in patients with partial recovery (13.78 months; range, 6–2) than in those with complete recovery (6.70 months; range, 3–12).

Fassler et al. reported on 7 patients with sciatic nerve injury involving both the tibial and peroneal divisions who had complete or nearly complete motor and sensory recovery of the tibial component but did not have satisfactory recovery of the peroneal component [12]. They attributed this finding to the degree of injury in the peroneal nerve division. The better recovery of the tibial division may reflect various factors, including anatomical location, blood supply, and the surrounding soft tissue [28]. In our present study, both the tibial and common peroneal nerve divisions were significantly improved after surgery. However, the extent of recovery of the common peroneal nerve division was not better than that of the tibial nerve division. Although the mean LSUHSC peripheral nerve score was lower for the common peroneal division than for the tibial division, the difference was not statistically significant.

The mean time taken for partial nerve recovery was longer than that required for complete recovery. However, there was no relationship between degree of recovery after sciatic nerve injury and sex, age, cause of injury, type of fracture, hip dislocation, and femoral head injury. The degree of nerve recovery is related to whether there is any abnormality in the nerve at the time of exploration. Neurological recovery was very good in our study, possible because none of the patients had severe neurological damage and follow-up was adequate. In view of the clinical characteristics of acetabular fractures combined with sciatic nerve injury, the sciatic nerve should be explored routinely during surgical treatment and measures taken that are conducive to correct determination of the prognosis and neurological recovery according to the type and degree of injury.

This study had several limitations. First, it had a retrospective design. Therefore, the possibility that the incidence of sciatic nerve injury in association with acetabular fracture was underrecognized cannot be excluded. Second, the study was performed at a single center and included a small sample, which is to be expected considering the relative rarity of such cases. Multicenter studies in larger populations are needed. Third, the peripheral nerve scoring system used in this study does not fully represent sensory and motor function, especially the sensory aspects, which are highly subjective and complex and may have affected physicians’ judgment. Fourth, as this is a retrospective study, MRI (magnetic resonance imaging), ultrasonography and EMG (electromyography) have not been used in these cases, which could be included in future study. Finally, our follow-up protocol did not fully cover factors such as rehabilitation exercises and physiotherapy, which may impact on the results.

## Conclusion

All traumatic sciatic nerve injuries associated with acetabular fractures involved the posterior wall or posterior column, and most patients had posterior dislocation of the hip joint. Damage to the common peroneal division was more severe than that to the tibial division before surgery. However, the degree of recovery of the common peroneal division was not worse than that of the tibial division; all patients had a recovery (completely or partially). The degree of neurological recovery was related to whether the nerve was found to be abnormal on intraoperative exploration. Patients with partial recovery required a longer recovery time.

## Data Availability

All the data are contained within this manuscript.

## References

[1] Laird A, Keating JF (2005). Acetabular fractures: a 16-year prospective epidemiological study. J Bone Jt Surg Br.

[2] Mauffrey C, Hao J, Cuellar DO, 3rd, et al. The epidemiology and injury patterns of acetabular fractures: Are the USA and China comparable? Clin Orthop Relat Res 2014;472(11):3332–7 10.1007/s11999-014-3462-8 PMC4182390 24442842

[3] Rinne PP, Laitinen MK, Huttunen T (2017). The incidence and trauma mechanisms of acetabular fractures: a nationwide study in Finland between 1997 and 2014. Injury.

[4] Lu S, Liu F, Xu W (2022). Management of open tile C pelvic fractures and their outcomes: a retrospective study of 30 cases. Ther Clin Risk Manag.

[5] Simske NM, Krebs JC, Heimke IM (2019). Nerve injury with acetabulum fractures: incidence and factors affecting recovery. J Orthop Trauma.

[6] Ahmed M, Abuodeh Y, Alhammoud A (2018). Epidemiology of acetabular fractures in Qatar. Int Orthop.

[7] Kanawati AJ, Narulla RS, Lorentzos P (2015). The change in position of the sciatic nerve during the posterior approach to the hip. Bone Jt J.

[8] Isin Y, Hapa O, Kara YS (2020). A CT study of the femoral and sciatic nerve periacetabular moving in different hip positions. J Orthop Surg Res.

[9] Kline DG, Kim D, Midha R (1998). Management and results of sciatic nerve injuries: a 24-year experience. J Neurosurg.

[10] Lehmann W, Hoffmann M, Fensky F (2014). What is the frequency of nerve injuries associated with acetabular fractures?. Clin Orthop Relat Res.

[11] Foulk DM, Mullis BH (2010). Hip dislocation: evaluation and management. J Am Acad Orthop Surg.

[12] Fassler PR, Swiontkowski MF, Kilroy AW (1993). Injury of the sciatic nerve associated with acetabular fracture. J Bone Jt Surg Am.

[13] Kreder HJ, Rozen N, Borkhoff CM (2006). Determinants of functional outcome after simple and complex acetabular fractures involving the posterior wall. J Bone Jt Surg Br.

[14] Mears DC, Velyvis JH, Chang CP (2003). Displaced acetabular fractures managed operatively: indicators of outcome. Clin Orthop Relat Res.

[15] Issack PS, Toro JB, Buly RL (2007). Sciatic nerve release following fracture or reconstructive surgery of the acetabulum. J Bone Jt Surg Am.

[16] Meccariello L, Razzano C, De Dominicis C (2021). A new prognostic pelvic injury outcome score. Med Glas.

[17] Stavrakakis IM, Kritsotakis EI, Giannoudis PV, et al. Sciatic nerve injury after acetabular fractures: a meta-analysis of incidence and outcomes. Eur J Trauma Emerg Surg. 2022;1–16. 10.1007/s00068-022-01896-0 35169868

[18] Judet R, Judet J, Letournel E (1965). Fractures of the acetabulum: classification and surgical approaches for open reduction. Preliminary report. J Bone Jt Surg.

[19] Letournel E, Judet R (2012). Fractures of the acetabulum.

[20] Issack PS, Helfet DL (2009). Sciatic nerve injury associated with acetabular fractures. HSS J.

[21] Letournel E, Judet R, Elson RA. Late complications of operative treatment within three weeks of injury. In: Fractures of the acetabulum. Springer; 1993. pp. 541–63.

[22] Tile M (1980). Fractures of the acetabulum. Orthop Clin N Am.

[23] Simske NM, Krebs JC, Heimke IM (2019). Nerve Injury with acetabulum fractures: incidence and factors affecting recovery. J Orthop Trauma.

[24] Gupta S, Mittal N, Virk JS (2018). Use of tricortical iliac crest strut autograft in comminuted posterior wall acetabular fractures: a case series. Chin J Traumatol.

[25] Iqbal F, Taufiq I, Najjad MK (2016). Fucntional and radiological outcome of surgical management of acetabular fractures in tertiary care hospital. Hip Pelvis.

[26] Magu NK, Gogna P, Singh A (2014). Long term results after surgical management of posterior wall acetabular fractures. J Orthop Traumatol.

[27] Liu X, Xu S, Zhang C (2010). Application of a shape-memory alloy internal fixator for treatment of acetabular fractures with a follow-up of two to nine years in China. Int Orthop.

[28] Kim DH, Murovic JA, Tiel R (2004). Management and outcomes in 353 surgically treated sciatic nerve lesions. J Neurosurg.

